# Probabilistic Relative Entropy in Homogenization of Fibrous Metal Matrix Composites (MMCs)

**DOI:** 10.3390/ma16186112

**Published:** 2023-09-07

**Authors:** Marcin Kamiński

**Affiliations:** Research Head of Civil Engineering, Geodesy & Transportation, Department of Structural Mechanics, Lodz University of Technology, 93-590 Lodz, Poland; marcin.kaminski@p.lodz.pl

**Keywords:** metal matrix composites, homogenization, Finite Element Method, Bhattacharyya relative entropy, response function method

## Abstract

The main aim of this work is to deliver uncertainty propagation analysis for the homogenization process of fibrous metal matrix composites (MMCs). The homogenization method applied here is based on the comparison of the deformation energy of the Representative Volume Element (RVE) for the original and for the homogenized material. This part is completed with the use of the Finite Element Method (FEM) plane strain analysis delivered in the ABAQUS system. The probabilistic goal is achieved by using the response function method, where computer recovery with a few FEM tests enables approximations of polynomial bases for the RVE displacements, and further—algebraic determination of all necessary uncertainty measures. Expected values, standard deviations, and relative entropies are derived in the symbolic algebra system MAPLE; a few different entropy models have been also contrasted including the most popular Kullback–Leibler measure. These characteristics are used to discuss the influence of the uncertainty propagation in the MMCs’ effective material tensor components, but may serve in the reliability assessment by quantification of the distance between extreme responses and the corresponding admissible values.

## 1. Introduction

As it is well known, metal matrix composites are composite materials having metal, alloy, or intermetallic matrices [1,2,3], which are reinforced with various types of fibers [4,5], particles [6], or whiskers [7]; layered sheets [8] are alternatively manufactured. Recently, some nanoparticle reinforcements have been invented [9], graphene reinforcements have been implemented [10], and hybrid MMCs have been designed [11]. From the material composition point of view, the MMCs are classified into titanium, magnesium, nickel, and copper-based metallic matrices, which offer quite different contrasts of the Young modulus of the matrix with respect to its reinforcement; aluminum matrices are also applicable to minimize the mass of such a composite. Typically, a volume fraction of the fibers reaches 30–40% of the composite, and one of the most popular reinforcing fibers is made of silicon carbide (SiC) [12] or carbon [13]; some alternative reinforcement with carbon nanostructures is also considerable. All these composite materials have been extensively studied in the last 20–30 years due to their wide and important applications in automotive and marine engineering, electronics, and aeronautics.

It is well-known that one of the most efficient mathematical and numerical techniques to analyze composite structures is the homogenization method. Interestingly, this method was and still is applicable to MMCs as the manufacturing method [14]. This numerical approach has been applied to investigate thermal stresses [15], MMCs reinforced with short fibers [16], and CNT/MMC under plastic deformation [17]. Additionally, the effective thermo-elastic characteristics were calculated [18], and an attempt to analyze MMCs with random fiber distribution has also been described [19]. Uncertainty analysis and quantification in the area of MMCs were rather scarce and have been focused on acoustic emission [20], damage detection and propagation [21], general reliability analysis [22], as well as strength prediction [23]. Analysis of photographic evidence of MMC cross-sections shows that the most important geometrical uncertainties needing attention and numerical simulation are the distribution of the reinforcement into the MMCs domain, interface shape, possible decohesion or lack of contact between the reinforcement and the matrix, as well as reinforcement diameter (and/or length). On the other hand, quite naturally, uncertainties are to be taken into account in mechanical, thermal, and electrical material parameters due to the manufacturing and processing of these composites in higher temperatures and difficulties in obtaining homogeneity within the MMC matrix.

Therefore, the main aim of this paper is to provide probabilistic homogenization [24,25] and uncertainty propagation analysis for some metal matrix composites by taking into account material imperfections within the matrix. Two different numerical techniques are engaged for this purpose, namely the iterative generalized higher-order stochastic perturbation method as well as the semi-analytical technique. They are implemented using numerical recovery of the polynomial bases [26] linking effective elasticity tensor components with the uncertainty source thanks to several FEMhomogenization tests delivered in the ABAQUS system. These techniques enable the determination of the basic probabilistic characteristics of the homogenized tensor such as expectations and standard deviations, while uncertainty propagation is studied thanks to different relative entropy models available from probability theory [27]. These include the Kullback–Leibler model [28], Bhattacharyya theory [29], Hellinger idea [30], as well as its Jeffreys symmetrization [31,32]. Relative entropy models can be efficiently used to quantify the distance between different pairs of random distributions, which can be represented in structural and computational mechanics by (1) effective and original characteristics of the composites, (2) extreme structural responses and their admissible counterparts (limit functions in the reliability assessment), as well as (3) computed structural stresses and deformations and their counterparts following full-scale experiments. The first case is studied in this paper in the context of all the most popular relative entropy mathematical models recalled above. It needs to be emphasized that all probabilistic mathematical tools together with the Weighted Least Squares Method (WLSM) polynomial bases recovery have been implemented in the computer algebra environment MAPLE, where numerical visualization has been completed. The main motivation of this research was to check how the contrast between the fiber and the matrix elastic moduli affects the probabilistic distance between original and homogenized characteristics of the MMC, which was tested on two different composites, namely MoSiO_2_-SiC and Ti-SiC [24,25]. Finally, it needs to be mentioned that this computer strategy may be extended towards thermo-mechanical homogenization of different MMCs, corrosion, and aging stochastic modeling, as well as numerical analysis of the interface defects appearing between various types of reinforcements and metallic matrices.

## 2. Homogenization Method

**Figure 1:**
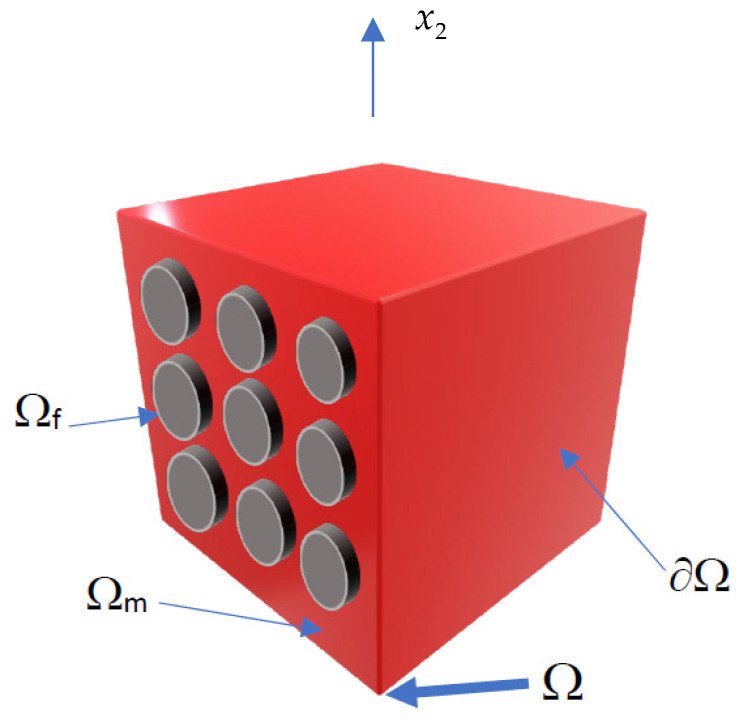
Schematic view of the fiber-reinforced composite RVE.

Effective material characteristics of various composites (such as fibrous or particulate, for instance) may be determined using an equation of deformation energies of real and homogenized equivalent structures. The corresponding numerical simulation, most frequently with the use of the Finite Element Method, takes place in the RVE of the given composite domain. Some specific Dirichlet boundary conditions imposed throughout all outer surfaces of this RVE represent uniaxial, biaxial, and transverse deformations, while periodicity conditions are fulfilled on the remaining ones; any von Neumann conditions apply. Using classical notation available in the literature, one writes this energy *U* (and its equation) as [27]

(1) $$U=\int_{\Omega }C_{ijkl}^{}\varepsilon_{ij}\varepsilon_{kl}\;d\Omega =\int_{\Omega }C_{ijkl}^{\left(eff\right)}{\bar{\varepsilon }}_{ij}{\bar{\varepsilon }}_{kl}\;d\Omega ,\mathrm{for}i,j,k,l=1,\;2,\;3$$

where Ω denotes the RVE (cf. Figure 1), $C_{ijkl}^{\left(eff\right)}$ and $C_{ijkl}^{}$ are the homogenized and the original composite constitutive tensor, $\varepsilon_{ij}$ stands for the strain tensor (adjacent to the adopted geometrical equations), and ${\bar{\varepsilon }}_{ij}$ represents unitary or zero strains depending on a specific $C_{ijkl}^{\left(eff\right)}$ component to be determined.

They follow the uniaxial, biaxial, as well as transverse kinematic boundary conditions defined on ∂Ω. The left-hand side integral is determined from the FEM tests in the presence of some uncertainty parameter (*b*) thanks to the following discretization of the displacements ($u_{i}=u_{i}\left(x_{i}\right)$) and strain tensor ($\varepsilon_{ij}=\varepsilon_{ij}\left(x_{i}\right)$) fields in Ω:

(2) $$u_{i}=\varphi_{i\alpha }q_{\alpha }=\varphi_{i\alpha }A_{\alpha }^{k}b^{k},\;\varepsilon_{ij}=\frac{1}{2}\left(u_{i,j}+u_{j,i}\right)=\frac{1}{2}\left(\varphi_{i\alpha ,j}+\varphi_{j\alpha ,i}\right)q_{\alpha }=B_{ij\alpha }q_{\alpha }=B_{ij\alpha }A_{\alpha }^{k}b^{k}\mathrm{for}i,j=1,\;2,\;3\;\&\;k=l,\ldots ,P,\alpha =l,\ldots ,N$$

where *P* denotes the order of polynomial basis on Ω, and *N* stands for the total number of degrees of freedom in the RVE. Let us note that stress tensor discretization is unnecessary to determine homogenized tensor in this approach and is omitted, but possible. Then, the fundamental FEM equations system with applied boundary conditions is solved as

(3) $$K_{\alpha \beta }\;q_{\beta }=0_{\alpha },\;K_{\alpha \beta }=\sum_{e=1}^{E}\int_{\Omega_{e}}B_{ij\alpha }^{T}C_{ijkl}^{\left(e\right)}B_{kl\beta }\;d\Omega \;\mathrm{for}i,j=1,\;2,\;3\;\&\;\alpha ,\beta =l,\ldots ,N$$

where $K_{\alpha \beta }$ stands for the global stiffness matrix of the RVE, *E* is the number of the finite elements in its discretization. A summation of the finite element contributions has no algebraic character except for the one adjacent to FEM global stiffness matrix assemblage; this is performed only once for the entire numerical homogenization process. Therefore, the component $C_{1111}^{\left(eff\right)}$ is determined from Equation (1) while assuming ${\bar{\varepsilon }}_{11}=1$ (extension with $\Delta_{1}$ and all the remaining components are set to 0), $C_{1122}^{\left(eff\right)}$—for ${\bar{\varepsilon }}_{11}=1$ & ${\bar{\varepsilon }}_{22}=1$ (biaxial tension using $\Delta_{1}$ and $\Delta_{2}$, zeroes elsewhere), and finally $C_{1212}^{\left(eff\right)}$—with ${\bar{\varepsilon }}_{12}=1$ only (shear of the RVE with $\Delta_{12}$). Finally:

(4) $$C_{1111}^{\left(eff\right)}=\frac{1}{\left\|\Omega \right\|}\int_{\Omega_{f}}C_{ijkl}^{}\varepsilon_{ij}\left(\Delta_{1}\right)\varepsilon_{kl}\left(\Delta_{1}\right)\;d\Omega +\frac{1}{\left\|\Omega \right\|}\int_{\Omega_{m}}C_{ijkl}^{}\varepsilon_{ij}\left(\Delta_{1}\right)\varepsilon_{kl}\left(\Delta_{1}\right)\;d\Omega$$

(5) $$C_{1122}^{\left(eff\right)}=\frac{1}{\left\|\Omega \right\|}\int_{\Omega_{f}}C_{ijkl}^{}\varepsilon_{ij}\left(\Delta_{1},\Delta_{2}\right)\varepsilon_{kl}\left(\Delta_{1},\Delta_{2}\right)\;d\Omega +\frac{1}{\left\|\Omega \right\|}\int_{\Omega_{m}}C_{ijkl}^{}\varepsilon_{ij}\left(\Delta_{1},\Delta_{2}\right)\varepsilon_{kl}\left(\Delta_{1},\Delta_{2}\right)\;d\Omega$$

(6) $$C_{1212}^{\left(eff\right)}=\frac{1}{\left\|\Omega \right\|}\int_{\Omega_{f}}C_{ijkl}^{}\varepsilon_{ij}\left(\Delta_{12}\right)\varepsilon_{kl}\left(\Delta_{12}\right)\;d\Omega +\frac{1}{\left\|\Omega \right\|}\int_{\Omega_{m}}C_{ijkl}^{}\varepsilon_{ij}\left(\Delta_{12}\right)\varepsilon_{kl}\left(\Delta_{12}\right)\;d\Omega$$

These relations include $\left\|\Omega \right\|$ as the total area (or volume in 3D) of the RVE, while $\Omega_{f}$ and $\Omega_{m}$ denote the domains occupied by the fiber and the matrix, correspondingly.

## 3. Probabilistic Governing Equations

Bhattacharyya’s relative entropy is a subject of further theoretical and numerical analysis. It quantifies the distance between the probability density functions of the original composite constitutive tensor and its homogenized counterpart. It is possible to calculate this relative entropy with the use of the Bhattacharyya theory as [29]

(7) $$H_{ijkl}=H\left(C_{ijkl}^{\left(eff\right)}\left(b\right),C_{ijkl}\left(b\right)\right)=\int_{-\infty }^{+\infty }{\left\{p\left(C_{ijkl}^{\left(eff\right)}\right)(x)\;p\left(C_{ijkl}\right)(x)\right\}}^{\frac{1}{2}}dx,\;\mathrm{for}i,j,k,l=1,2,3$$

for any subset $\Omega_{i}$ of Ω. Some referential models invented by Kullback and Leibler, Jeffreys, and Hellinger have been recalled here:

(8) $$H_{KL}(p,q)=-\int_{-\infty }^{+\infty }p(x)\log(q(x))dx+\int_{-\infty }^{+\infty }q(x)\log(p(x))dx,$$

(9) $$H_{J}(p,q)=H_{KL}\left(p,q\right)+H_{KL}\left(q,p\right),$$

(10) $$H_{SH}(p,q)=\frac{1}{2}\int_{-\infty }^{+}{\left(\sqrt{p(x)}-\sqrt{q(x)}\right)}^{2}dx=1-\int_{-\infty }^{+\infty }\sqrt{p(x)q(x)}\;dx$$

One derives them in the case of two non-truncated Gaussian distributions representing the original fourth-order elasticity tensor ($p\left(\mu \left(C_{ijkl}\right),\sigma \left(C_{ijkl}\right)\right)$) and the effective tensor ($q\left(\mu \left(C_{ijkl}^{\left(eff\right)}\right),\sigma \left(C_{ijkl}^{\left(eff\right)}\right)\right)$) [28,29,30,31,32]:

(11) $$H_{KL}\left(C_{ijkl},C_{ijkl}^{\left(eff\right)}\right)=\log\left(\frac{\sigma \left(C_{ijkl}^{\left(eff\right)}\right)}{\sigma \left(C_{ijkl}\right)}\right)+\frac{\sigma_{}^{2}\left(C_{ijkl}\right)+{\left(\mu \left(C_{ijkl}\right)-\mu \left(C_{ijkl}^{\left(eff\right)}\right)\right)}^{2}}{2\sigma_{}^{2}\left(C_{ijkl}^{\left(eff\right)}\right)}-\frac{1}{2}$$

(12) $$\begin{array}{l} H_{J}(C_{ijkl},C_{ijkl}^{\left(eff\right)})=\log\left(\frac{\sigma \left(C_{ijkl}^{\left(eff\right)}\right)}{\sigma \left(C_{ijkl}\right)}\right)+\frac{\sigma_{}^{2}\left(C_{ijkl}\right)+{\left(\mu \left(C_{ijkl}\right)-\mu \left(C_{ijkl}^{\left(eff\right)}\right)\right)}^{2}}{2\sigma_{}^{2}\left(C_{ijkl}^{\left(eff\right)}\right)}\\ \;\;\;\;\;\;\;\;+\log\left(\frac{\sigma \left(C_{ijkl}\right)}{\sigma \left(C_{ijkl}^{\left(eff\right)}\right)}\right)+\frac{\sigma_{}^{2}\left(C_{ijkl}^{\left(eff\right)}\right)+{\left(\mu \left(C_{ijkl}^{\left(eff\right)}\right)-\mu \left(C_{ijkl}\right)\right)}^{2}}{2\sigma_{}^{2}\left(C_{ijkl}\right)}-1 \end{array}$$

(13) $$H_{H}\left(C_{ijkl},C_{ijkl}^{\left(eff\right)}\right)=1-\sqrt{\frac{2\sigma \left(C_{ijkl}\right)\sigma \left(C_{ijkl}^{\left(eff\right)}\right)}{\sigma_{}^{2}\left(C_{ijkl}\right)+\sigma_{}^{2}\left(C_{ijkl}^{\left(eff\right)}\right)}}\;\exp\left(-\frac{1}{4}\frac{{\left(\mu \left(C_{ijkl}\right)-\mu \left(C_{ijkl}^{\left(eff\right)}\right)\right)}^{2}}{\sigma_{}^{2}\left(C_{ijkl}\right)+\sigma_{}^{2}\left(C_{ijkl}^{\left(eff\right)}\right)}\right)$$

(14) $$H_{B}\left(C_{ijkl},C_{ijkl}^{\left(eff\right)}\right)=\frac{1}{4}\frac{{\left(\mu \left(C_{ijkl}^{\left(eff\right)}\right)-\mu \left(C_{ijkl}\right)\right)}^{2}}{\sigma_{}^{2}\left(C_{ijkl}^{\left(eff\right)}\right)+\sigma_{}^{2}\left(C_{ijkl}\right)}+\frac{1}{2}\ln\left(\frac{\sigma_{}^{2}\left(C_{ijkl}^{\left(eff\right)}\right)+\sigma_{}^{2}\left(C_{ijkl}\right)}{2\sigma \left(C_{ijkl}\right)\sigma \left(C_{ijkl}^{\left(eff\right)}\right)}\right)$$

A very important case, taking into account the results of further numerical simulation, is when the homogenized tensor is a linear transform of a normally distributed matrix Young modulus, i.e.,

(15) $$C_{ijkl}^{\left(eff\right)}=A_{1}^{ijkl}E_{m}+A_{0}^{ijkl}$$

Then, the first two probabilistic moments of the effective tensor components equal to

(16) $$E\left[C_{ijkl}^{\left(eff\right)}\right]=A_{1}^{ijkl}E\left[E_{m}\right]+A_{0}^{ijkl},\sigma \left(C_{ijkl}^{\left(eff\right)}\right)=A_{1}^{ijkl}\sigma \left(E_{m}\right)$$

Further, one derives the following algebraic equations for the aforementioned relative entropies where no summation of *i*,*j*,*k*, or *l* applies:

(17) $$H_{B}\left(C_{ijkl},C_{ijkl}^{\left(eff\right)}\right)=\frac{1}{4}\frac{{\left(\left(1-A_{1}^{ijkl}\right)E\left[E_{m}\right]-A_{0}^{ijkl}\right)}^{2}}{Var\left(E_{m}\right)\left(1+{\left(A_{1}^{ijkl}\right)}^{2}\right)}+\frac{1}{2}\log\left(\frac{1+{\left(A_{1}^{ijkl}\right)}^{2}}{2A_{1}^{ijkl}}\right)$$

(18) $$H_{KL}\left(C_{ijkl},C_{ijkl}^{\left(eff\right)}\right)=\frac{\log\left({\left(A_{1}^{ijkl}\right)}^{2}\right)}{2}+\frac{Var\left(E_{m}\right)+{\left(\left(1-A_{1}^{ijkl}\right)E\left[E_{m}\right]-A_{0}^{ijkl}\right)}^{2}}{2Var\left(E_{m}\right){\left(A_{1}^{ijkl}\right)}^{2}}-\frac{1}{2}$$

(19) $$\begin{array}{l} H_{J}(C_{ijkl},C_{ijkl}^{\left(eff\right)})=\frac{Var\left(E_{m}\right)+{\left(\left(1-A_{1}^{ijkl}\right)E\left[E_{m}\right]-A_{0}^{ijkl}\right)}^{2}}{2Var\left(E_{m}\right){\left(A_{1}^{ijkl}\right)}^{2}}\\ \;\;\;\;\;\;\;\;\;\;\;\;\;\;\;\;\;+\frac{Var\left(E_{m}\right){\left(A_{1}^{ijkl}\right)}^{2}+{\left(\left(1-A_{1}^{ijkl}\right)E\left[E_{m}\right]-A_{0}^{ijkl}\right)}^{2}}{2Var\left(E_{m}\right)}-1 \end{array}$$

(20) $$H_{H}\left(C_{ijkl},C_{ijkl}^{\left(eff\right)}\right)=1-\sqrt{\frac{A_{1}^{ijkl}}{1+{\left(A_{1}^{ijkl}\right)}^{2}}}\;\exp\left(-\frac{1}{4}\frac{{\left(A_{1}^{ijkl}E\left[E_{m}\right]+A_{0}^{ijkl}-E\left[E_{m}\right]\right)}^{2}}{Var\left(E_{m}\right)\left(1+{\left(A_{1}^{ijkl}\right)}^{2}\right)}\right)$$

It needs to be mentioned that all of these formulas include a square of the difference between the expectations of the original elasticity tensor components and their homogenized counterparts. This makes the measurement of the relative entropies of the probabilistic distance more sensitive to this difference than, for instance, the First Order Reliability Method (FORM) distance, where linear interrelation is employed.

## 4. Numerical Analysis and Discussion

**Figure 2:**
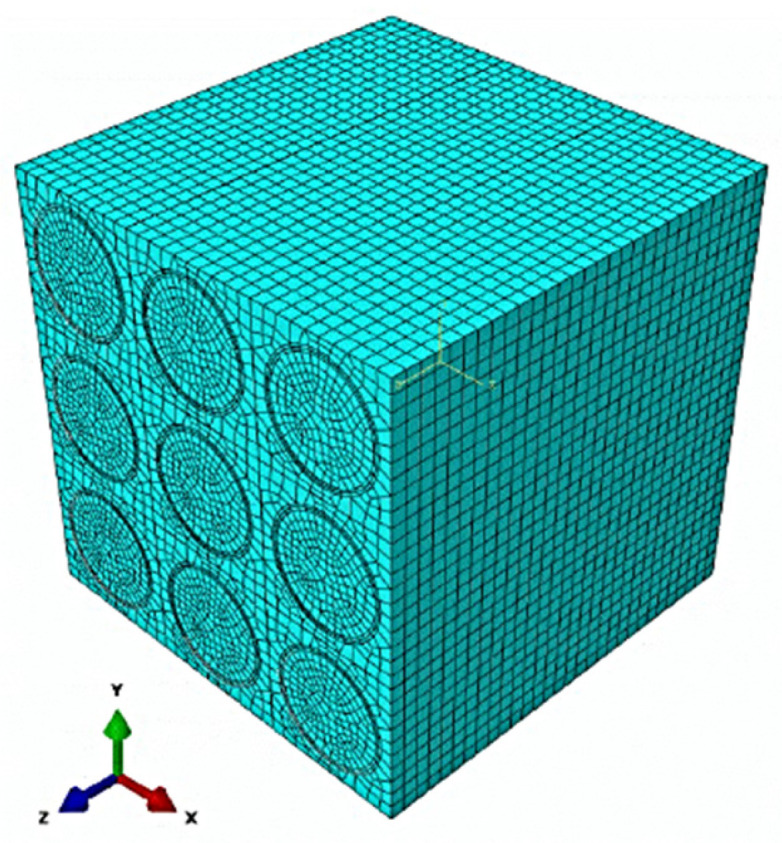
Discretization of the MMC Representative Volume Element (RVE).

The Finite Element Method discretization of the RVE consisting of nine parallel and uniformly distributed fibers has been completed in the ABAQUS system; some alternative FEM discretizations and studies can be found in [33,34]. It consists of 107.838 C3D8 hexagonal finite elements (with a 2 × 2 × 2 integration scheme), where the matrix includes 72.090 bricks, and the fiber—35.748 finite elements, cf. Figure 2. This RVE model has been subjected in turn to uniaxial extension in the *x* direction, to biaxial in the *x* and *y* directions (according to notation adopted automatically by the ABAQUS system in Figure 2, which corresponds to *x*_1_ and *x*_2_ from Figure 1), and also to shear deformation in the *xy* plane to remain in the plane strain state each time. Mean values of material characteristics of the two composites under investigation have been collected in Table 1, where the Young moduli of the matrix have been randomized accordingly. Two sets of initial deterministic FEM analyses have been delivered for the following discrete values of these moduli: (1) E_m_ = {360, 370, 380, 390, 400, 410, 420, 430, 440, 450} GPa, and also (2) E_m_ = {101.80, 104.80, 107.80, 110.80, 113.80, 116.80, 119.80, 122.80, 125.80} GPa.

**Figure 3:**
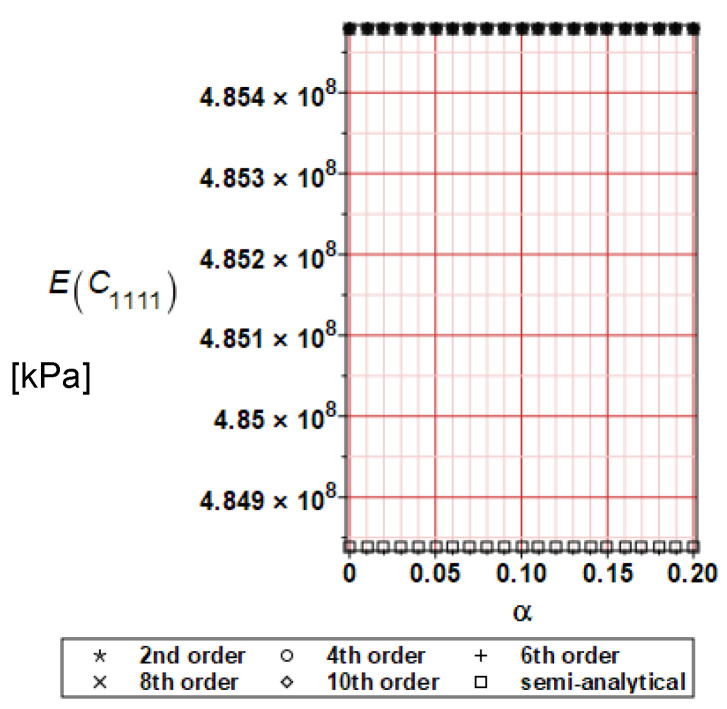
Expected values of C_1111_ for MoSiO_2_-SiC composite.

**Figure 4:**
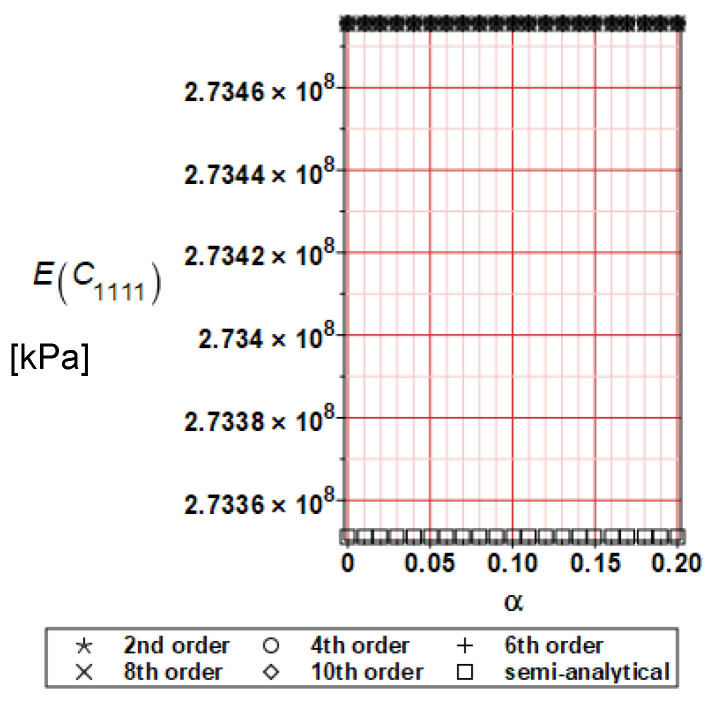
Expected values of C_1111_ for Ti-SiC composite.

**Figure 5:**
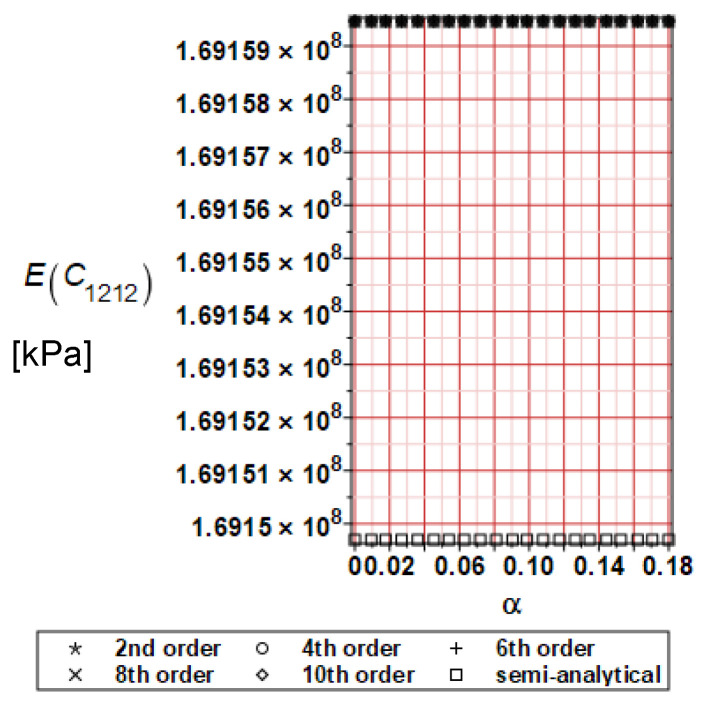
Expected values of C_1212_ for MoSiO_2_-SiC composite.

**Figure 6:**
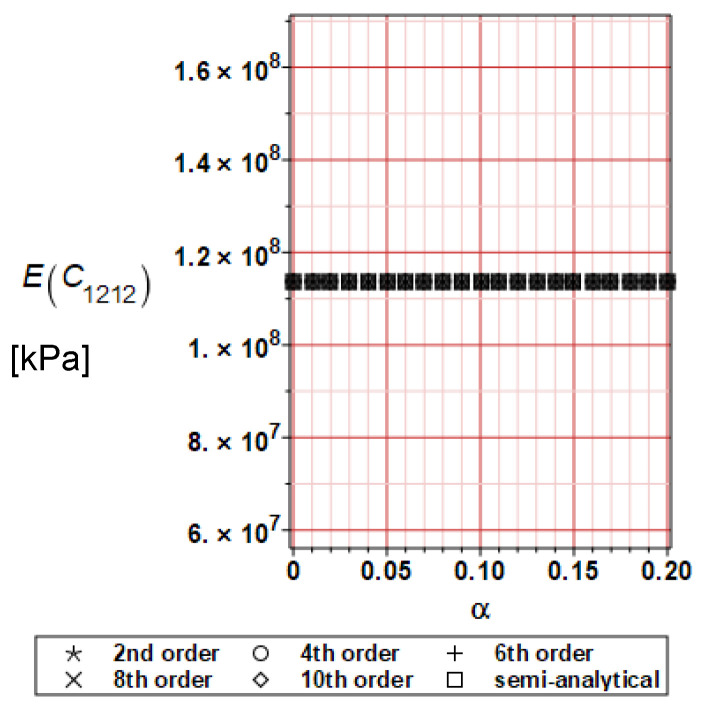
Expected values of C_1212_ for Ti-SiC composite.

**Figure 7:**
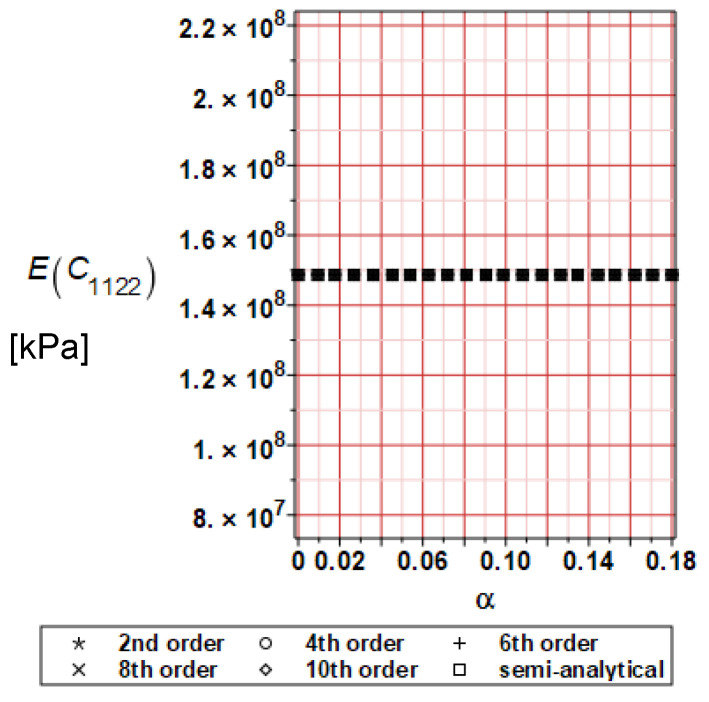
Expected values of C_1122_ for MoSiO_2_-SiC composite.

**Figure 8:**
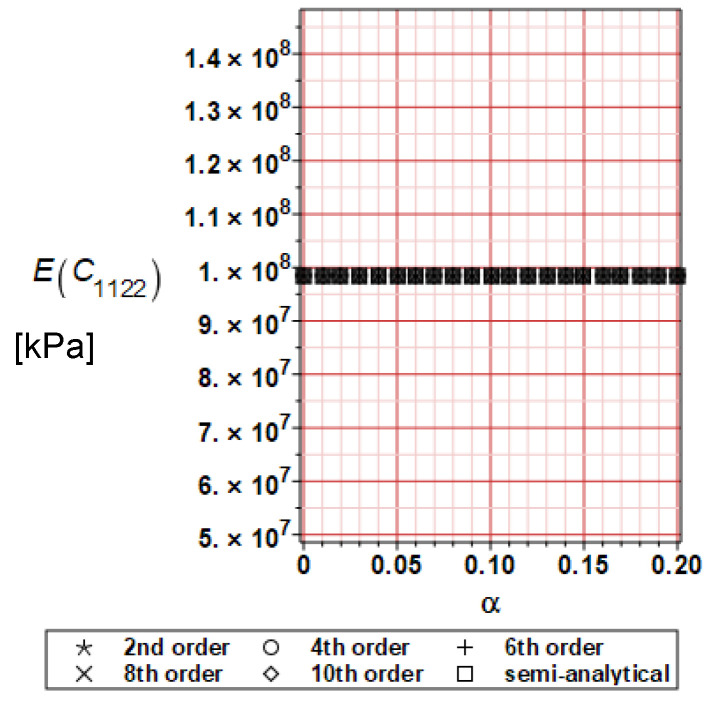
Expected values of C_1122_ for Ti-SiC composite.

**Figure 9:**
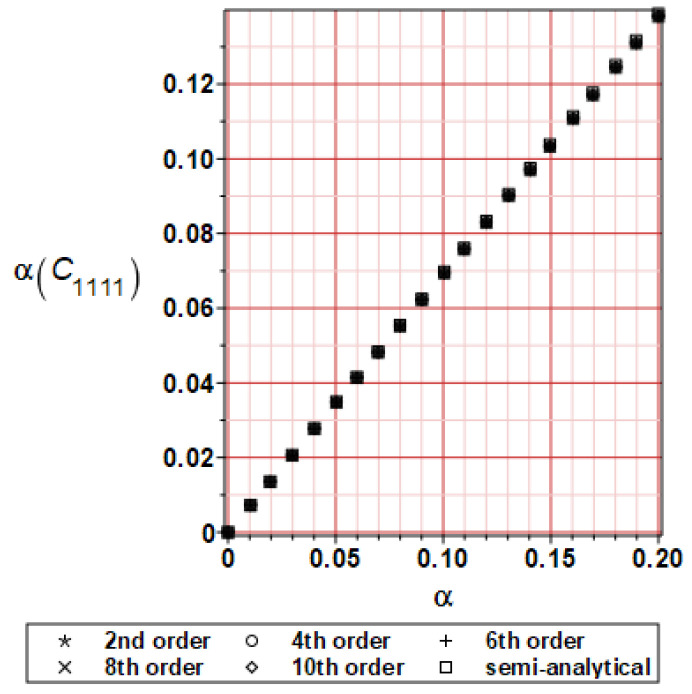
CoV of C_1111_ for MoSiO_2_-SiC composite.

**Figure 10:**
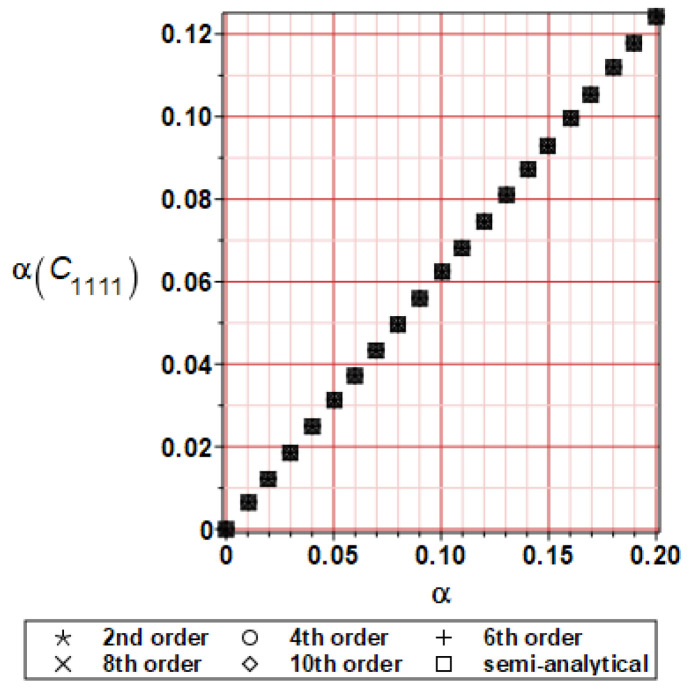
CoV of C_1111_ for Ti-SiC composite.

**Figure 11:**
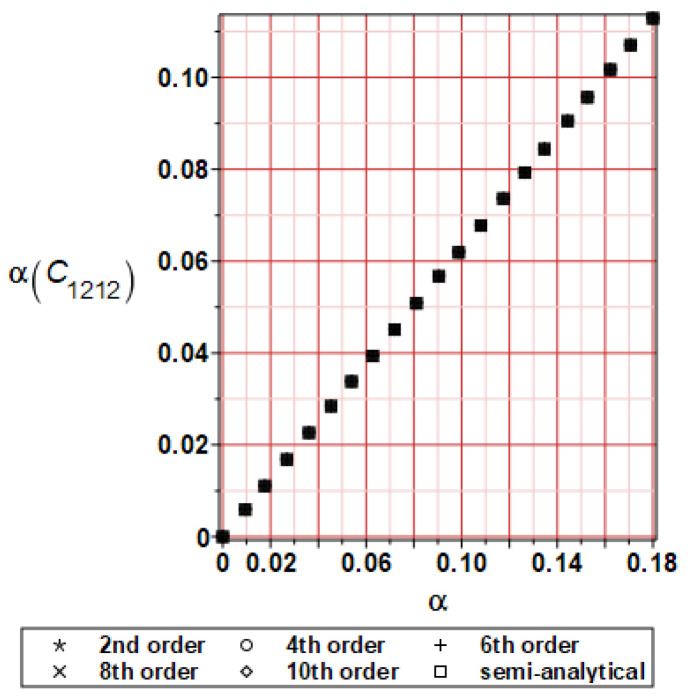
CoV of C_1212_ for MoSiO_2_-SiC composite.

**Figure 12:**
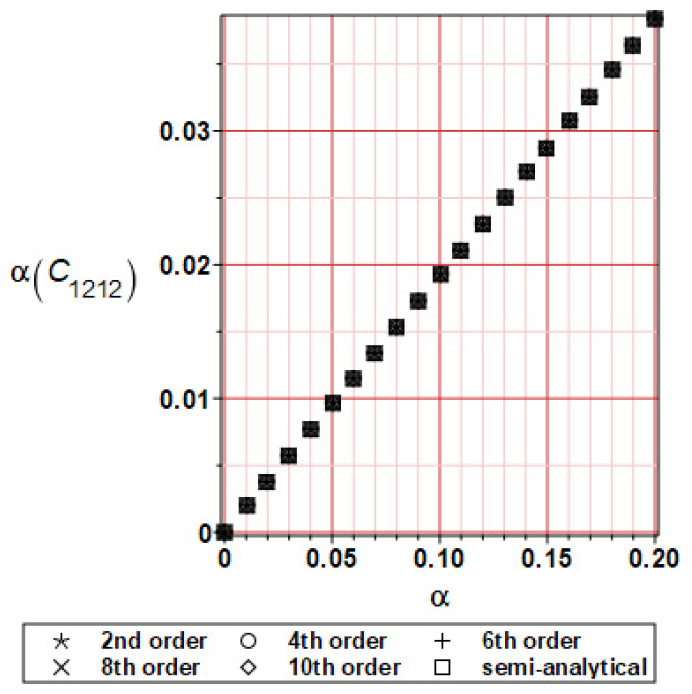
CoV of C_1212_ for Ti-SiC composite.

**Figure 13:**
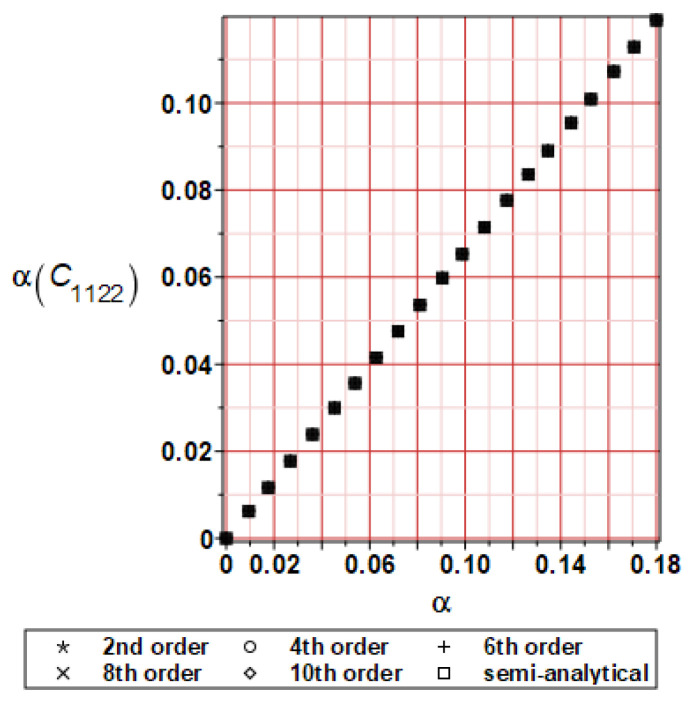
CoV of C_1122_ for MoSiO_2_-SiC composite.

**Figure 14:**
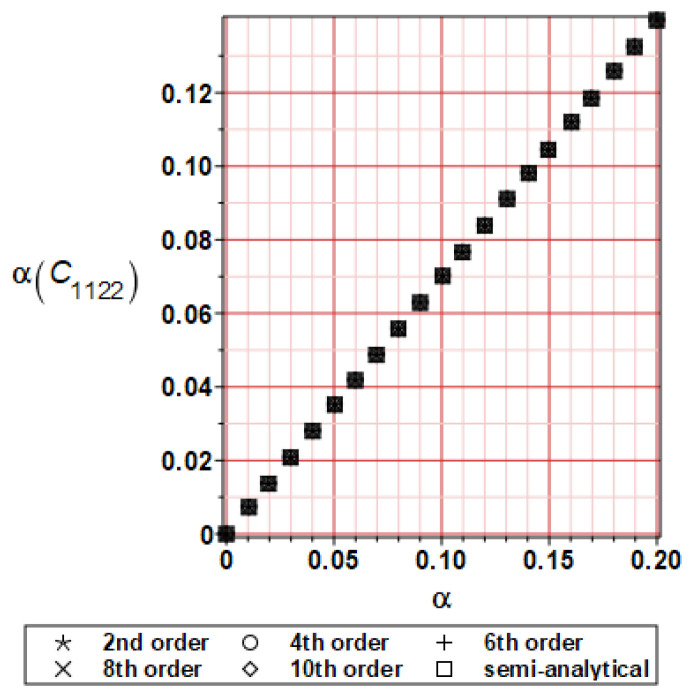
CoV of C_1122_ for Ti-SiC composite.

**Figure 15:**
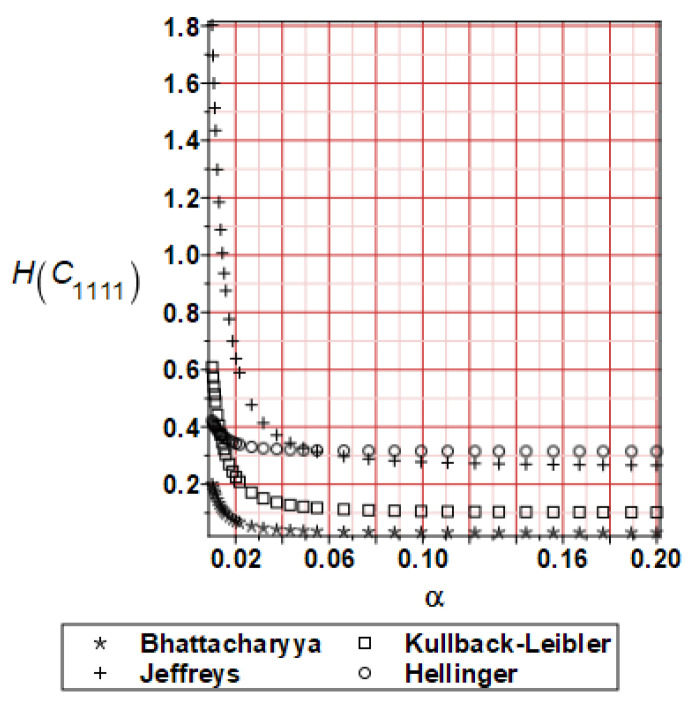
Relative entropies of C_1111_ for MoSiO_2_-SiC composite.

**Figure 16:**
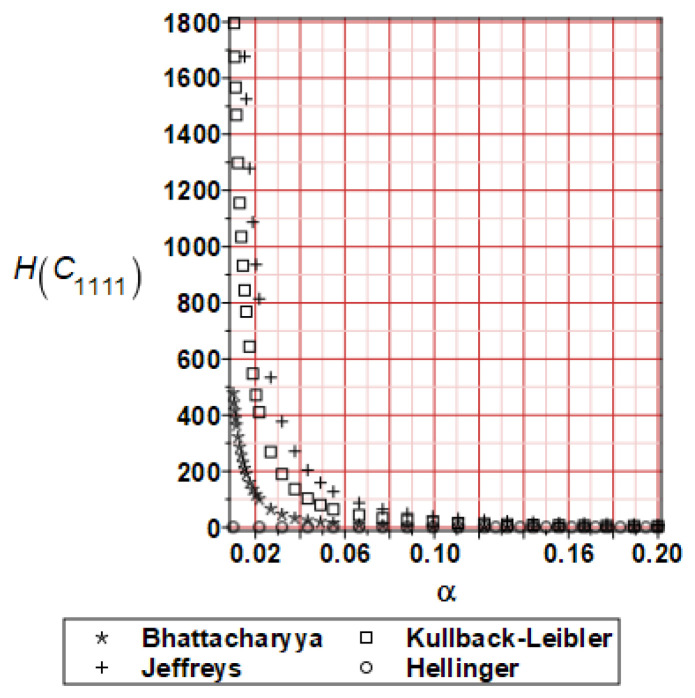
Relative entropies of C_1111_ for Ti-SiC composite.

**Figure 17:**
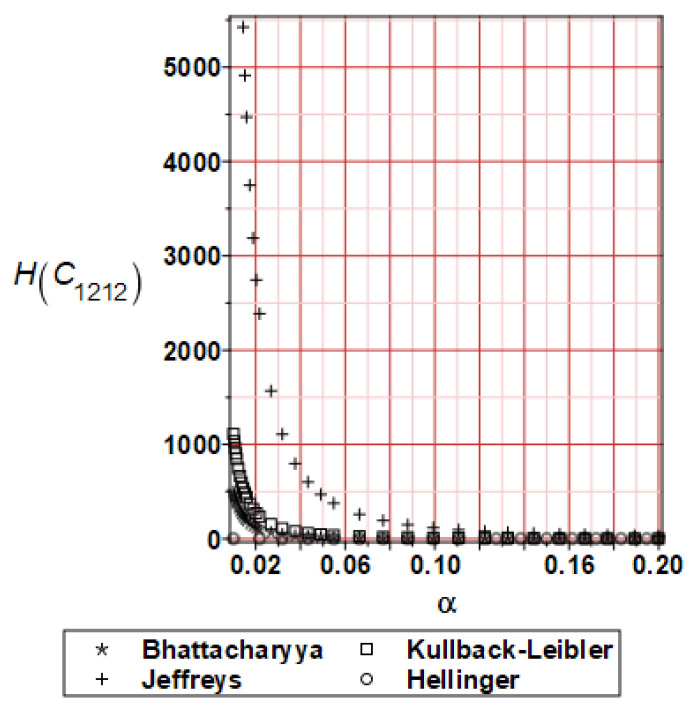
Relative entropies of C_1212_ for MoSiO_2_-SiC composite.

**Figure 18:**
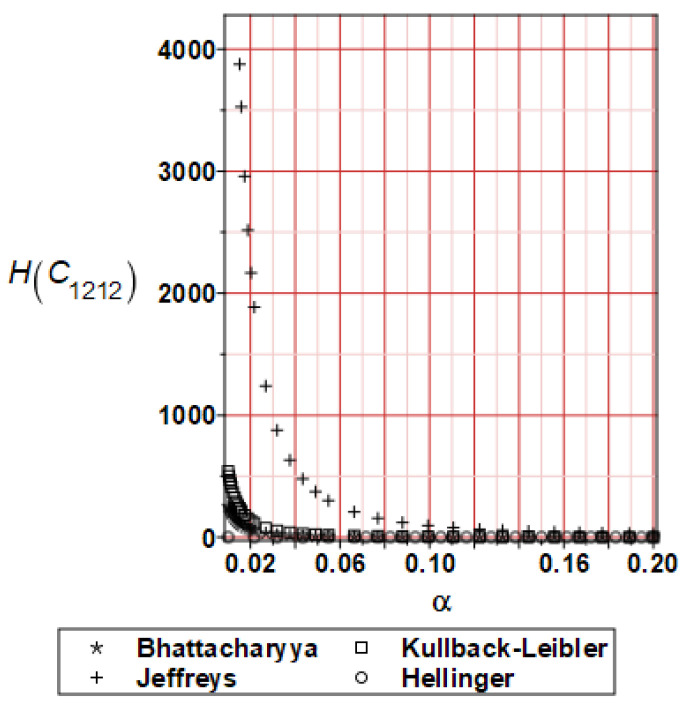
Relative entropies of C_1212_ for Ti-SiC composite.

**Figure 19:**
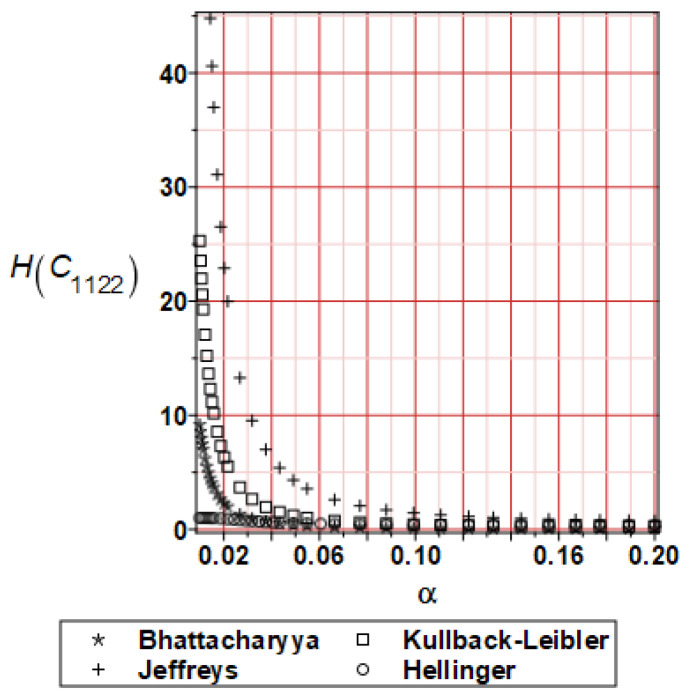
Relative entropies of C_1122_ for MoSiO_2_-SiC composite.

**Figure 20:**
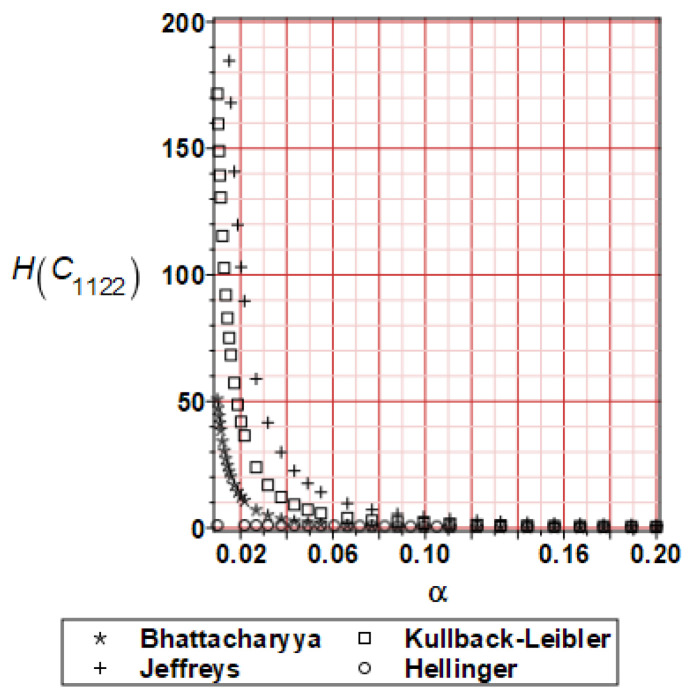
Relative entropies of C_1122_ for Ti-SiC composite.

It has been detected with the Weighted Least Squares Method that polynomial bases linking effective elasticity tensor components with the Young modulus of the metal matrix have a linear form. It has been demonstrated for two MMCs having essentially different contrasts of E_f_ and E_m_. This linearity follows a relatively large stiffness of the matrix in MMCs compared to the polymer-based fibrous composites, where polynomial bases have apparently curvilinear character. Therefore, the Second Order Second Moment (SOSM) analysis is sufficient, the Monte–Carlo simulation is unnecessary, and the semi-analytical approach returns simple and exact equations for the moments and the entropies of the homogenized tensor. Therefore, the Gaussian distribution of the Young modulus of the matrix implies Gaussian distribution of the effective tensor and this observation justifies the equations relevant to the relative entropy collected in the previous section. This is a very important result, which remarkably simplifies probabilistic homogenization as no higher moments are necessary and, further, relative entropies have all elegant closed-form algebraic formulas. It is seen that the mechanically driven and polynomial response-based approach is the most efficient in the homogenization of random composites. Probabilistic results of numerical simulations have been collected in Figure 3, Figure 4, Figure 5, Figure 6, Figure 7, Figure 8, Figure 9, Figure 10, Figure 11, Figure 12, Figure 13, Figure 14, Figure 15, Figure 16, Figure 17, Figure 18, Figure 19 and Figure 20 in two columns, where the left column corresponds to the MoSiO_2_-SiC composite (with smaller contrast), while the right column corresponds to the Ti-SiC composite. They include expected values (Figure 3, Figure 4, Figure 5, Figure 6, Figure 7 and Figure 8) and coefficients of variation (CoV, Figure 9, Figure 10, Figure 11, Figure 12, Figure 13 and Figure 14) of the homogenized tensor as well as relative entropies of this tensor in Figure 15, Figure 16, Figure 17, Figure 18, Figure 19 and Figure 20. These characteristics have been computed for the components C_1111_, C_1212,_ and C_1122_—all as the functions of the CoV of the Young modulus for the matrix $\alpha \in \left[0.00,0.20\right]$. The first two moments have been determined using the 2nd, 4th, 6th, 8th, and 10th order perturbation theories and with the semi-analytical technique. Relative entropies computed here contain the models created by Kullback and Leibler, Bhattacharyya, Hellinger, and Jeffreys.

The first general observation is that all probabilistic methods return exactly the same first two probabilistic moments for any value of the input α. Additionally, all expectations are constant for any input α, while the resulting CoVs of the effective tensor are linearly dependent on input uncertainty for both composites. Further, as is expected after the data collected in Table 1, expectations of the homogenized tensor for the MoSiO_2_-SiC composite are larger than the corresponding characteristics of the Ti-SiC composite; additionally, it has been noted that $E\left[C_{1111}^{\left(eff\right)}\right]$ > $E\left[C_{1122}^{\left(eff\right)}\right]$ > $E\left[C_{1212}^{\left(eff\right)}\right]$ in both cases. Almost the same conclusion holds true in the case of $\alpha \left(C_{ijkl}^{\left(eff\right)}\right)$ excluding the $C_{1122}^{\left(eff\right)}$ component uncertainty, where the resulting CoV for the Ti-SiC composite prevails. It is apparent that the resulting uncertainty in the homogenized characteristics is a little bit smaller than the input one. A ratio of this input-to-output uncertainty is not larger than two, with an exception for $C_{1212}^{\left(eff\right)}$ in the case of the Ti-SiC composite, where it is about five. Moreover, while contrasting the CoVs plotted in the left and in the right columns, it is clear that larger uncertainty propagation in homogenization due to Young modulus randomness is noticed for the MMC composite, where $E_{f}≅E_{m}$.

The final set of graphs shows a distance of *E_m_* probability distribution to $C_{ijkl}^{\left(eff\right)}$ PDFs for both composite materials. It is evident that all these entropies dramatically decrease while increasing the input CoV. This agrees very well with the fact that increasing these two given PDF ranges makes their distance smaller for a constant difference in their expectations. Moreover, it is documented here that various entropy models return quite different intervals of numerical values. The largest results are obtained while applying the Jeffreys model, smaller numbers are returned with the Kullback–Leibler theory, which is justified by their definitions, followed by Bhattacharyya entropy, and, finally, the Hellinger model; somewhat different relations are exceptionally obtained in the case of the very small distance depicted in Figure 15. Comparing the results included in both columns, one notices that the *E_m_* distribution is close to $C_{1111}^{\left(eff\right)}$ for the first composite with smaller contrast (*E_m_* almost equal *E_f_*). In the case of $C_{1212}^{\left(eff\right)}$, relative entropies are smaller for the second composite, where *E_m_*/*E_f_* ≈ 4, but the differences are not so huge as in the first case. The component $C_{1122}^{\left(eff\right)}$ exhibits some similarity to the behavior of $C_{1111}^{\left(eff\right)}$, where the first composite’s entropy is about four times smaller than the Ti-SiC composite. Summarizing the results concerning relative entropy obtained here, larger uncertainty propagation is expected for the metal matrix composite with smaller contrast between the elastic moduli of this fiber-reinforced composite. The resulting uncertainty in the MMCs’ effective characteristics starts to decrease when this contrast increases, and this is demonstrated with an increasing distance of original and homogenized characteristics. Further randomization of Poisson ratios, which is of secondary importance in the MMCs area, may bring larger differences between different probabilistic methods due to its nonlinear impact on the homogenized characteristics.

Let us also note that the relative entropies computed above follow the previous results concerning the first two probabilistic moments so that computational cost has been minimized by an application of the stochastic perturbation technique. This cost is closer to the deterministic FEM solution rather than to the full Monte Carlo simulation results, whereas its accuracy remains the same as for statistical simulations. In summary, it is clearly seen from this analysis that relative probabilistic entropy may be efficient in probabilistic sensitivity analysis, where a smaller distance between two distributions relating some input parameter and the output state function of the given composite is equivalent to a larger impact of this input on the resulting composite’s response.

## 5. Concluding Remarks

(1) It has been documented in this work that uncertainty analysis in the homogenization of the MMCs brings stable basic probabilistic moments of the effective tensor, which have been computed with minimal time and computer power effort. Uncertainty propagation while randomizing matrix elastic modulus reaches its maximum with *E_m_* ≈ *E_f_* (for composites with a small contrast between the matrix and the fiber) and decreases while increasing the contrast between these material parameters. Extending this study beyond the limits of the MMCs, rather small uncertainty should accompany homogenized characteristics for the polymer-based composites, where the largest uncertainty is usually observed for the polymers themselves.
(2) Relative entropies calculated here due to different mathematical models have enabled the study of the distance between the PDF for *E_m_* and the effective tensor components, but the Jeffreys model appeared to be the most distant to the rest of the numerical results. Nevertheless, the determination of these entropies makes it possible to discuss the probabilistic sensitivity of $C_{ijkl}^{\left(eff\right)}$ components of the given MMC as a function of the aforementioned contrast. This contrast has been discovered as the most influential designing parameter in uncertainty propagation for the given class of composites, so the application of the apparatus presented for the optimization of composite constituents seems to be reasonable and promising. It is documented that future computations of all uncertainty measures for the MMCs may be performed in the future with the lower-order stochastic perturbation method as the fastest approach having the same accuracy as semi-analytical methods, as far as the uncertainty in Young moduli would be considered. This method is relatively easily applicable to any FEM system, contrary to the semi-analytical approach, where symbolic calculus plays a decisive role. The main difficulty while discussing relative entropies for uncertainty of homogenized characteristics is a lack of reference values in the literature and the fact that different theories may return separate numerical value ranges.
(3) Further extensions of this model towards uncertainty quantification in the homogenization process for the nonlinear composites or multi-materials structures subjected to stochastic aging would enable for numerical simulation of some technologically important processes, including some failure and/or corrosion. An application of the stochastic kriging technique [35] or polynomial chaos [36] may be a good alternative to the methods presented above when cross-correlations of multiple uncertainty sources would be expected. An application of the XFEM approach [37] could be beneficial for geometrical randomness homogenization (especially through a few geometrical scales), whereas the probability transformation method (PTM) may enable faster determination of probabilistic (relative) entropy [38].

## Figures and Tables

**Table 1:** Material characteristics of the MMCs.

No.	Composite	Matrix		Fiber	
		E_m_ [GPa]	ν_m_	E_f_ [GPa]	ν_f_
1	MoSiO_2_-SiC	400	0.25	450	0.20
2	Ti-SiC	113.80	0.33	450	0.20

## Data Availability

The data contained in this paper are available upon the special request sent to the Author.
